# Do free caesarean section policies increase inequalities in Benin and Mali?

**DOI:** 10.1186/s12939-018-0789-x

**Published:** 2018-06-05

**Authors:** Marion Ravit, Martine Audibert, Valéry Ridde, Myriam De Loenzien, Clémence Schantz, Alexandre Dumont

**Affiliations:** 1 IRD (French Institute For Research on sustainable Development), CEPED (IRD-Université Paris Descartes), Universités Paris Sorbonne Cités, ERL INSERM SAGESUD, Paris, France; 2 Université Clermont Auvergne, CNRS, CERDI, F-63000 Clermont-Ferrand, France; 3 Institut de Recherche en Santé Publique de Montréal (IRSPUM), Montréal, Canada; 4 Centre Population et Développement (CEPED), UMR 196 IRD-Université Paris Descartes, 45 rue des Saints-Pères, 75006 Paris, France

**Keywords:** Caesarean section, Low-income countries, Mali, Benin, Health policy, User fees, Health equity, Maternal health

## Abstract

**Background:**

Benin and Mali introduced user fee exemption policies focused on caesarean sections (C-sections) in 2005 and 2009, respectively. These policies had a positive impact on access to C-sections and facility based deliveries among all women, but the impact on socioeconomic inequality is still highly uncertain. The objective of this study was to observe whether there was an increase or a decrease in urban/rural and socioeconomic inequalities in access to C-sections and facility based deliveries after the free C-section policy was introduced.

**Methods:**

We used data from three consecutive Demographic and Health Surveys (DHS): 2001, 2006 and 2011–2012 in Benin and 2001, 2006 and 2012–13 in Mali. We evaluated trends in inequality in terms of two outcomes: C-sections and facility based deliveries. Adjusted odds ratios were used to estimate whether the distributions of C-sections and facility based deliveries favoured the least advantaged categories (rural, non-educated and poorest women) or the most advantaged categories (urban, educated and richest women). Concentration curves were used to observe the degree of wealth-related inequality in access to C-sections and facility based deliveries.

**Results:**

We analysed 47,302 childbirths (23,266 in Benin and 24,036 in Mali). In Benin, we found no significant difference in access to C-sections between urban and rural women or between educated and non-educated women. However, the richest women had greater access to C-sections than the poorest women. There was no significant change in these inequalities in terms of access to C-sections and facility based deliveries after introduction of the free C-section policy.

In Mali, we found a reduction in education-related inequalities in access to C-sections after implementation of the policy (*p*-value = 0.043). Inequalities between urban and rural areas had already decreased prior to implementation of the policy, but wealth-related inequalities were still present.

**Conclusions:**

Urban/rural and socioeconomic inequalities in C-section access did not change substantially after the countries implemented free C-section policies. User fee exemption is not enough. We recommend switching to mechanisms that combine both a universal approach and targeted action for vulnerable populations to address this issue and ensure equal health care access for all individuals.

## Background

The number of maternal deaths has decreased worldwide since 1990; however, 275,000 women still died giving birth in 2015, and almost half of these deaths occurred in sub-Saharan Africa [1]. A systematic review concluded that poorer women or women living in rural areas have less access to skilled delivery than richer women or those living in urban areas [2]. Inequalities also exist in terms of access to caesarean sections (C-sections), a major life-saving intervention needed by 3.6 to 6.5% of pregnant women [3]. Although C-section rates have increased in most low- and middle-income countries, these rates have typically increased faster for women in the richest quintile than for those in the poorest one [4]. A recent study of 2003–2013 showed that C-sections were extremely rare (less than 1%) among rural poorer women in seven out of 11 studied West and Central African countries, and only one country had a C-section rate higher than 2%. In contrast, eight of these countries had C-section rates higher than 4% among richer urban women [5].

In the 1980s, many African countries introduced user fees at the point of service to improve the quality of health services and access to primary health care. This change was supported by the Bamako Initiative (BI), which aimed to promote community financing of health services [6, 7]. User fees at the point of health service became a major barrier to health care access, especially for vulnerable populations [8]. This barrier is especially relevant for maternal health [9]. The fear of having to pay for excessive expenses may even lead some women to deter or delay their decision to seek care when they are dealing with obstetric complications [10]. Because C-sections are an expensive intervention, their access is directly influenced by household wealth and other non-financial factors [11], and the procedure can lead to catastrophic expenses [12, 13].

Some African countries have implemented user fee exemption policies for maternal health care services to improve access to maternal health services. In the 2000s, Benin and Mali, two western African countries with very high maternal mortality rates (respectively 405 and 587 per 100,000 live births), a low human development index ranking (167 and 175 out of 188,) and a high fertility rate (4.9 and 6.4 births per women) [14], decided to remove user fees only for women who receive a C-section.

In April 2009, the government of Benin introduced a national user fee exemption policy concerning all C-sections in selected public and private hospitals that offer emergency obstetric care. Hospitals receive 100,000 CFA (US$166) per C-section, which covers pre-operative laboratory tests, medications, surgery kits, blood, hospitalization for 7 days and transportation to a hospital if the woman is referred [15, 16]. The state is the principal financer of the reform [16].

The government of Mali introduced a user fee removal reform on January 1, 2005; this reform concerns all C-sections in the public sector and covers the surgical procedure and pre-operative examinations, the surgical kit and postoperative treatment (a standardized set of products and medications), and hospitalization. Structures receive 30,000 FCFA (US$50) for a simple C-section and 42,000 FCFA (US$70) for a complicated C-section in addition to a surgical kit [17, 18].

In contrast to other financing mechanisms, such as conditional cash transfers or targeted vouchers, the fee exemption policy for C-sections in Mali and Benin concerns all women and does not target poorer, rural or non-educated women. This policy has had a positive impact on access to C-sections and facility based delivery (FBD) among favoured and unfavoured women [19]. The impact of user fee exemptions on socioeconomic inequality is still highly uncertain [20]. In Mali, a patient survey study conducted 5 years after implementation of the free C-section policy to estimate the distribution of C-sections across socioeconomic groups showed that wealthier women clearly had greater access to C-sections than poorer women [21]. Some studies showed that user fee removal was far from sufficient to ensure equity in access to maternal health care, and in some cases, it increased existing inequality in access [22–26].

Two recent studies used a robust approach via the difference-in-differences method to assess the effects of pregnancy-related fee removal on inequalities in access to maternal health services in Ghana, Senegal, Kenya and Burkina Faso [22, 27]. Only one of these studies evaluated the impact of the fee exemption policy on inequalities in access to C-section and found that the reform had its greatest impact on rural and less educated women [27]. However, this study did not determine whether inequalities were reduced after the policy was implemented in the studied countries compared to countries without a fee exemption policy.

The aim of this study was to observe whether there was an increase or a decrease in urban/rural and socioeconomic inequalities in access to C-sections and facility based deliveries after the free C-section policy was introduced in Mali and Benin.

## Methods

We observed the evolution of inequalities in access to C-sections and facility based deliveries in the two countries through an observational study using repeated cross-sectional surveys.

### Data available

We selected three Demographic and Health Surveys (DHSs) in each country covering a period over 15 years: (1) Benin: 2001, 2006 and 2011–2012 and (2) Mali: 2001, 2006 and 2012–13.

DHSs are funded by the U.S. Agency for International Development (USAID) and have been conducted approximately every 4 or 5 years in more than 90 countries (https://dhsprogram.com) since the beginning of the 1980s. These household surveys are nationally representative with large sample sizes (usually between 5000 and 30,000 households) that provide a wide range of information on, for example, child health, education, domestic violence, HIV prevalence and maternal health. These surveys are free and available on demand.

In DHSs, interviews were conducted with women aged 15 to 49 years old who spent the night before the interview in the surveyed household. Women were interviewed on their pregnancies in the last 5 years prior the survey. We selected information on the last birth for each of the surveyed women (if a woman delivered more than one child during this period, we selected the last birth only). Data on household characteristics (demographic, socioeconomic and environmental conditions) and on the last pregnancy, including information on the use of maternal health services, were extracted from relevant questionnaires. During the interview, the woman was asked whether her child was born by C-section. As recommended, for greater accuracy, C-section cases among births that occurred at home were recoded as vaginal birth deliveries [28]. Furthermore, on the DHSs, socioeconomic status was evaluated using principal component analysis [29, 30]. The wealth index is a composite measure of a household’s cumulative living standard. The calculation is based on the household conditions and assets, such as televisions, telephones, vehicles, materials used for housing construction, and types of water access and sanitation facilities. The wealth index is calculated separately for each survey, which allows measuring the wealth of each household relative to others. For each woman, we used the household relative wealth index and wealth quintile available in each DHS survey.

### Measures and exposure variables

We studied the trends in inequalities in women’s access to C-sections and FBD in Benin and Mali. C-sections were the primary outcome because we assumed that policy implementation had a direct positive impact on women’s access to C-sections. FBD was chosen as a secondary outcome to assess whether the policy helped improve access healthcare facilities. An FBD was defined as a birth that occurred in a public or private healthcare facility (hospital, health centre or clinic). Other births (taxi, car or home) were coded as non-FBD.

We studied the evolution of inequalities in terms of zone of residence, education and wealth. Zone of residence is a binary variable (urban or rural) that corresponds to the areas of residence of the woman during the interview. Concerning education, we considered a woman as educated if she received at least a primary education. Finally, wealth was measured by the wealth index described below and the wealth quintile (from the poorest to the richest).

Other variables used in the analysis were the maternal age by category (< 18 years, 18–34 years, and 35 years or more), parity (primipara or multipara) and the number of newborns (singleton pregnancy or multiple pregnancy).

**Fig. 1:**
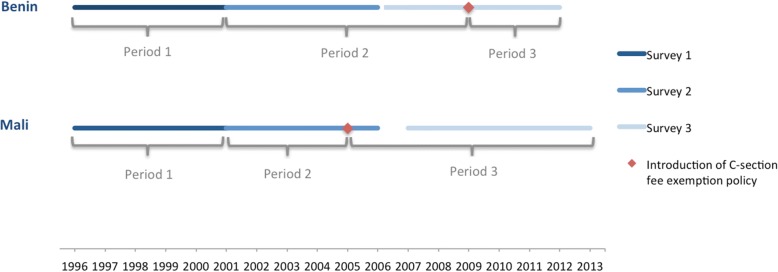
Dates of birth data available by country and survey. DHS surveys (http://dhsprogramme.com/). C-section = caesarean section. In Benin: Period 1 = September 1996 to November 2001; Period 2 = December 2001 to March 2009; and Period 3 = April 2009 to March 2012. In Mali: Period 1 = February 1996 to May 2001; Period 2 = June 2001 to December 2004; and Period 3 = January 2005 to January 2013

We identified three study periods according to the policy introduction date in each country (Fig. 1). Study periods 1 and 2 corresponded to births before implementation of the free C-section policy, and study period 3 corresponded to the period afterwards. Period 1 included all births that occurred within 5 years prior to DHS 2001 in both countries. Since the policy was implemented in 2005 in Mali, period 2 included only the births that occurred from June 2001 to December 2004 prior to DHS 2006. The other births that occurred after December 2004 were included in period 3. For the same reason, and since the policy was implemented in 2009 in Benin, period 2 in Benin included births from DHS 2006 and DHS 2011–12 that occurred from December 2001 to April 2009. Period 3 in Benin included only births that occurred from April 2009 to March 2012 prior to DHS 2011–12.

### Statistical analysis

To study the trends in inequalities, we performed a multivariate logistic regression with a two-way interaction (time*categories) using the following form:

$$ Logit\left[P\left({Y}_{igt}\right)\right]=\alpha +\delta {category}_i+\gamma {time}_i+\beta group\ast {category}_i+{X}_i $$

where *Y* is an indicator of whether woman *i* gave birth by C-section; *category* is a dummy variable indicating whether the woman belongs to the least or most advantaged category; *time* is a dummy variable indicating whether the birth occurred before (period 1 or 2) or after adoption of the policy (period 3); and *Xict* is a vector of individual-level covariates.

In this case, *β* measures whether the change in inequalities regarding access to C-section rates between the most and the least advantaged categories is significantly different between periods (i.e., *p*-value of the interaction test is < 0.05). Multivariate logistic regression models were adjusted based on maternal age, education, zone of residence, wealth quintile of household, parity, and multiple pregnancy, as well as sampling weight, clustering and strata. We analysed two outcomes (C-sections and FBD) and two types of inequalities (urban-rural and education-related inequalities) for each country. For each of the logistic regressions, we performed a Hosmer-Lemeshow goodness-of-fit test, which allowed us to assess the model fit after fitting a logistic regression model taking survey design into account [31].

Adjusted odds ratios (aORs) associated with *β* from these regression were used to measure urban-rural and socioeconomic inequalities in access to C-sections. We first used an aOR, which is commonly applied in health and social sciences to measure inequalities [32, 33]. The OR here represents the odds of having a C-section (proportion of women who had a C-section divided by the proportion of women who did not have a C-section) in the least advantaged categories (rural, non-educated or poorest) divided by the odds of having a C-section in the most advantaged category (urban, educated or richest). An OR below 1 indicates inequalities in favour of the least advantaged categories (they are more likely to have C-section than the most advantaged categories), a value over 1 indicates inequalities in favour of the most advantaged categories (they are more likely to have a C-section than the least advantaged categories), and an OR equal to 1 indicates an equal distribution of C-sections among all women. Using logistic regression models, we calculated aORs on variables selected a priori as potentially affecting C-sections (area of residence, maternal age, education level, wealth quintile of household, parity, multiple pregnancy) and considered sampling weight, clustering and strata.

Third, to complete our analyses of socioeconomic inequalities, we used concentration curves (CCs) [34, 35] to present the degree of socioeconomic inequality in access to C-section. CCs plot the cumulative percentage of C-section rates (y-axis) and the cumulative percentage of women ranked by household wealth index (available in the DHS survey) in the order of poorest to richest women (x-axis). If every woman, regardless of her wealth status, has the same access to C-section, the CC is a 45-degree line running from the bottom left-hand corner to the top right-hand corner, called the line of equity. If the CC is above the line of equality, it means that C-sections are more concentrated among the poor than among the rich. By contrast, if the CC is below the line of equity, the richest women have greater access to C-sections than the poorest women. The closer the CC is to the line of equity, the less important the inequalities in access to C-section are. We performed a CC by period for each country. We tested whether the concentration index (area under the curve) was significantly different between periods (Z-test).

No imputation of missing data was performed*.* Tests were two-tailed, and *p* < 0.05 was considered statistically significant. We managed the data with SPSS version 20 (SPSS Inc., Chicago, IL), and analyses were performed using Stata version 13.0 software (Stata Corp., College Station, TX, USA).

## Results

We used the three DHSs for each country to analyse 47,302 women who delivered a live-born child in the 5 years prior to the interview (23,266 women in Benin and 24,036 women in Mali).

**Fig. 2:**
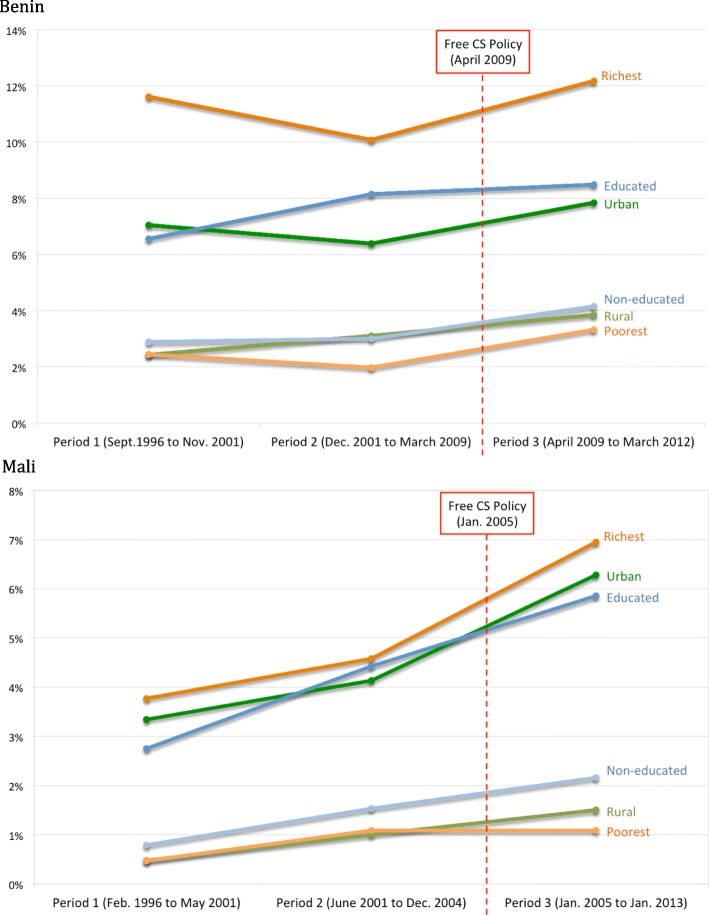
Trends in C-section rates in Benin and Mali by category and period**.** DHS surveys (http://dhsprogram.com/). CS = caesarean section. In Benin: Period 1 = September 1996 to November 2001; Period 2 = December 2001 to March 2009; and Period 3 = April 2009 to March 2012. In Mali: Period 1 = February 1996 to May 2001; Period 2 = June 2001 to December 2004; and Period 3 = January 2005 to January 2013

Figure 2 illustrates the evolution of C-section rates between study periods in Benin and Mali. In these two countries, regardless of the period, the C-section rates were higher for women in the most advantaged categories (urban, educated or richest) than for those in the other categories.

Table 1 presents the characteristics of women by period and country.

**Table 1:** Characteristics of pregnant women^a^ by period in Benin and Mali

	Benin			Mali		
	Period 1	Period 2	Period 3	Period 1	Period 2	Period 3
	N *(%)*	N *(%)*	N *(%)*	N *(%)*	N *(%)*	N *(%)*
	3616	12,809	6841	8280	4166	11,590
Age categories						
< 18 yr.	48 (1.3)	140 (1.1)	105 (1.5)	241 (2.9)	57 (1.4)	460 (4.0)
18–34 yr.	2634 (72.8)	9297 (72.6)	5359 (78.3)	5914 (71.4)	2799 (67.2)	8696 (75.0)
35 yr. and more	934 (25.8)	3372 (26.3)	1377 (20.1)	2125 (25.7)	131 (31.5)	2434 (21.0)
Parity						
Primipara	729 (20.1)	2206 (17.2)	1368 (20.0)	1344 (16.2)	655 (15.7)	2029 (17.5)
Multipara	2887 (79.8)	10,603 (82.8)	5473 (80.0)	6936 (83.8)	3511 (84.3)	9561 (82.5)
Multiple pregnancy						
Singleton	351 (97.0)	12,403 (96.8)	6673 (97.5)	8132 (98.2)	4093 (98.3)	11,386 (98.2)
Multiple	106 (2.9)	406 (3.2)	168 (2.5)	148 (1.8)	73 (1.8)	204 (1.8)
Education level						
None	2637 (72.9)	9515 (74.3)	4943 (72.3)	6960 (84.1)	3465 (83.2)	9486 (81.9)
Primary or more	979 (27.1)	3294 (25.7)	1898 (27.7)	1320 (15.9)	701 (16.8)	2104 (18.2)
Zone of residence						
Urban	1138 (31.5)	4744 (37.0)	2577 (37.7)	1801 (21.8)	1357 (32.6)	3216 (27.8)
Rural	2478 (68.5)	8065 (63.0)	4264 (62.3)	6479 (78.3)	2809 (67.4)	8374 (72.3)
Wealth quintiles of households						
Q1 Poorest	816 (22.6)	2850 (22.2)	1533 (22.4)	1706 (20.6)	731 (17.6)	2211 (19.1)
Q2 Poorer	782 (21.6)	2623 (20.5)	1473 (21.5)	1641 (19.8)	813 (19.5)	2353 (20.3)
Q3 Middle	752 (20.0)	2675 (20.9)	1448 (21.2)	1854 (22.4)	903 (21.7)	2341 (20.2)
Q4 Richer	697 (19.3)	2584 (20.2)	1326 (19.4)	1720 (20.8)	887 (21.3)	2324 (20.1)
Q5 Richest	569 (15.7)	2077 (16.2)	1061 (15.5)	1359 (16.4)	832 (20.0)	2361 (20.4)
Place of delivery						
Home or other	814 (22.5)	2432 (19.0)	870 (12.7)	5193 (62.7)	2202 (52.9)	5367 (46.3)
Public facility	2347 (64.9)	8676 (67.7)	5210 (76.2)	2979 (36.0)	1890 (45.4)	5941 (51.3)
Private facility	447 (12.4)	1689 (13.2)	743 (10.9)	76 (0.9)	66 (1.6)	275 (2.4)
Missing	8 (0.2)	12 (0.1)	18 (0.3)	32 (0.4)	8 (0.2)	7 (0.1)
Delivery by C-section						
No	3474 (96.1)	12,208 (95.3)	6471 (94.6)	8156 (98.5)	4079 (97.9)	1126 (97.2)
Yes	140 (3.9)	552 (4.3)	366 (5.4)	91 (1.1)	84 (2.0)	328 (2.8)
Missing	2 (0.1)	49 (0.4)	4 (0.1)	33 (0.4)	3 (0.1)	2 (0.0)

Source: DHS surveys (http://dhsprogram.com/)

C-section = caesarean section

In Benin: Period 1 = September 1996 to November 2001; Period 2 = December 2001 to March 2009; and Period 3 = April 2009 to March 2012

In Mali: Period 1 = February 1996 to May 2001; Period 2 = June 2001 to December 2004; and Period 3 = January 2005 to January 2013

^a^Women who delivered a live-born child within 5 years prior to the interview

The number of pregnant women differs greatly by period and country because the births from the study periods do not correspond to the births from the DHSs. Indeed, the policy introduction date is shifted in time: 2005 in Mali and 2009 in Benin. This difference in timing explains why there are more studied births in period 2 than in period 3 in Benin and why these results contrast with the results for Mali.

The mode of delivery was available for 99.8% of the included women (47,209 women), and the place of delivery was available for 99.8% of the included women (47,217 women).

### Benin

Table 2 shows that in Benin, there is no significant inequality in access to C-section between urban and rural women or between educated and non-educated women, irrespective of the period. However, the results revealed significant inequality in access to FBD between educated and non-educated women in period 1 (adjusted OR = 1.58; 95% CI 1.21 to 2.05), and this inequality increased between periods 1 and 2 before implementation of the C-section fee exemption policy and between periods 1 and 3 (*p*-value = 0.001). There are significant poorest-richest inequalities in access to C-sections and FBD but no significant change after the introduction of the policy.

**Table 2:** Rate of delivery by C-section and in a facility for the most recent birth of each woman within 5 years prior to their interview in Benin

	Before implementation of the policy		After implementation of the policy	Trends in inequalities between period 1 and period 3
	Period 1	Period 2	Period 3	*p*-value^c^
Total				
All pregnant women	3614	12,760	6837	
% delivery by C-section	3.87	4.33	5.35	
% FBD	77.44	81.00	87.25	
Women by zone of residence				
Urban	1137	4723	2574	
% delivery by C-section	7.04	6.39	7.85	
% FBD	85.44	86.97	92.02	
Rural	2477	8037	4263	
% delivery by C-section	2.42	3.11	3.85	
% FBD	73.78	77.48	84.37	
Urban/rural inequalities				
aOR (95% CI) for C-section^a^	1.44 (0.88; 2.35)	1.18 (0.96; 1.45)	1.24 (0.92; 1.66)	0.135
aOR (95% CI) for FBD^b^	0.75 (0.47; 1.21)	0.85 (0.65; 1.12)	0.97 (0.70; 1.35)	0.244
Women by level of education				
Educated	977	3280	1896	
% delivery by C-section	6.55	8.14	8.49	
% FBD	90.79	95.53	97.36	
Non-educated	2637	9480	4941	
% delivery by C-section	2.88	3.01	4.15	
% FBD	72.48	75.97	83.36	
Educated/non-educated inequalities				
aOR (95% CI) for C-section^a^	1.02 (0.71; 1.48)	1.87 (1.50; 2.33)***	1.21 (0.92; 1.59)	0.665
aOR (95% CI) for FBD^b^	1.58 (1.21; 2.05)**	3.31 (2.71; 4.06)***	3.13 (2.27; 4.33)***	0.001
Women by wealth quintiles of households (Q1 and Q5)				
Q1 - Poorest	816	2842	1532	
% delivery by C-section	2.45	1.97	3.33	
% FBD	56.81	60.57	72.17	
Q5 - Richest	568	2073	1060	
% delivery by C-section	11.62	10.08	12.17	
% FBD	98.24	98.41	99.24	
Poorest/richest inequalities				
aOR (95% CI) for C-section^a^	4.08 (1.96; 8.43)***	3.01 (1.96; 4.64)***	2.86 (1.69; 4.83)***	0.487
aOR (95% CI) for FBD^b^	58.63 (23.09148.89)***	35.02 (21.52; 57.01)***	37.57 (16.55; 85.28)***	0.525

*aOR* adjusted odds ratio: *C-section* caesarean section, and *FBD* facility based delivery

Period 1 = September 1996 to November 2001; Period 2 = December 2001 to March 2009; and Period 3 = April 2009 to March 2012

*p ≤ 0.05; **p ≤ 0.01; and ***p ≤ 0.001

^a^The aORs for C-section access were estimated with the use of multivariate logistic regression models (adjusted based on age, education, wealth quintile of household, zone of residence, parity, and multiple pregnancy, as well as sampling weight, clustering and strata)

^b^The aORs for FBD access were estimated with the use of multivariate logistic regression models (adjusted based on age, education, wealth quintile of household, zone of residence, parity, and multiple pregnancy, as well as sampling weight, clustering and strata)

^c^*p*-values of the interaction between categories (urban vs rural or educated vs non-educated) and period (period 1 vs period 3) were estimated with the use of the multivariate logistic regression models (adjusted based on age, education, wealth quintile of household, parity, and multiple pregnancy, as well as sampling weight, clustering and strata)

**Fig. 3:**
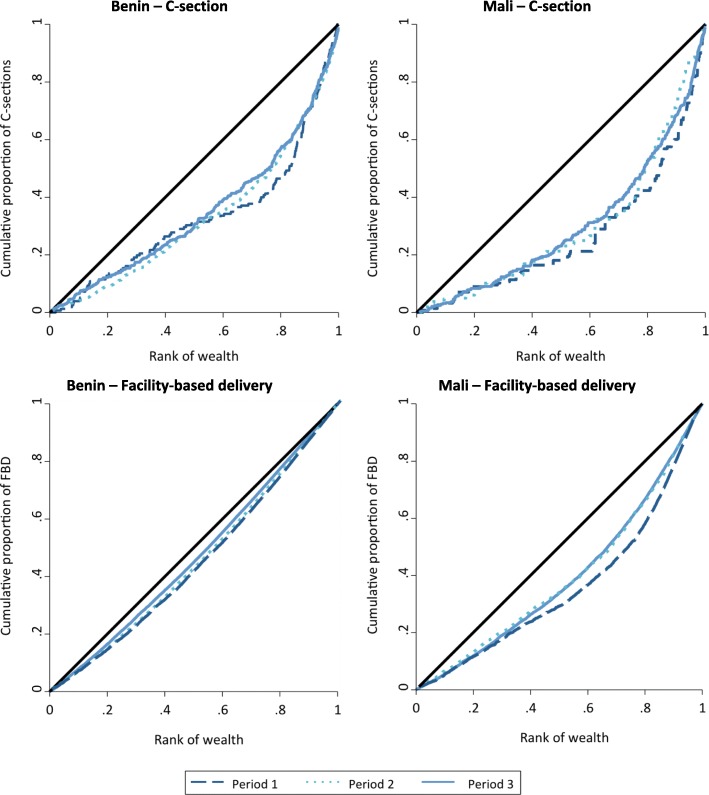
Concentration curves for C-section and facility based delivery in Mali and Benin between the late 1990s and early 2010s. FBD: facility based delivery In Benin: Period 1 = September 1996 to November 2001; Period 2 = December 2001 to March 2009; and Period 3 = April 2009 to March 2012. In Mali: Period 1 = February 1996 to May 2001; Period 2 = June 2001 to December 2004; and Period 3 = January 2005 to January 2013

Figure 3 shows that the CCs are below the line of equity irrespective of the outcome or period. Thus, our results confirm that the richest women have greater access to C-sections and FBD than the poorest women do. The results indicated no significant change between periods in wealth-related inequalities in access to C-section or FBD. In particular, the concentration index for C-sections in period 1 was not significantly different from the concentration index in period 3 (*p*-value of the Z-test = 0.322). A significant reduction in inequalities in FBD access was found between periods 1 and 3 (*p*-value = 0.000), but this decrease had already started prior to implementation of the policy.

### Mali

In period 1, the probability of having a C-section was four times higher for urban women than for rural women in Mali (Table 3). Inequalities in favour of urban women were still significant in period 3 but were halved after implementation of the policy (*p*-value = 0.032). Notably, these disparities started to decline in the early 2000s, prior to implementation of the policy. Urban-rural inequalities in access to FBD were also significant, but no significant change was found between periods 1 and 3.

**Table 3:** Rate of delivery by C-section and in a facility for the most recent birth of each woman within 5 years prior to their interview in Mali

	Before implementation of the policy		After implementation of the policy	Trends in inequalities between period 1 and period 3
	Period 1	Period 2	Period 3	*p*-value^c^
Total				
All pregnant women	8247	4163	11,588	
% delivery by C-section	1.10	2.02	2.83	
% FBD	37.04	47.04	53.66	
Women by zone of residence				
Urban	1794	1355	3215	
% delivery by C-section	3.34	4.13	6.28	
% FBD	80.42	79.57	87.15	
Rural	6453	2808	8373	
% delivery by C-section	0.48	1.00	1.50	
% FBD	24.99	31.30	40.80	
Urban/rural inequalities				
aOR (95% CI) for C-section^a^	4.68 (2.09; 10.46)***	1.96 (0.58; 6.61)	2.05 (1.40; 3.00)***	0.032
aOR (95% CI) for FBD^b^	4.37 (2.93; 6.53)***	3.22 (2.14; 4.86)***	2.60 (1.91; 3.55)***	0.121
Women by level of education				
Educated	1310	701	2103	
% delivery by C-section	2.75	4.42	5.85	
% FBD	64.74	80.54	81.53	
Non-educated	6937	3462	9485	
% delivery by C-section	0.79	1.53	2.16	
% FBD	31.80	40.27	47.49	
Educated/non-educated inequalities				
aOR (95% CI) for C-section^a^	2.01 (1.23; 3.30)**	2.02 (0.99; 4.14)*m	1.39 (1.02; 1.89)*	0.043
aOR (95% CI) for FBD^b^	2.18 (1.74; 2.72)***	3.68 (2.57; 5.29)***	2.06 (1.74; 2.43)***	0.702
Women by wealth quintiles of households (Q1 and Q5)				
Q1 - Poorest	1702	731	2211	
% delivery by C-section	0.47	1.09	1.09	
% FBD	21.16	29.26	30.76	
Q5 - Richest	1353	830	2360	
% delivery by C-section	3.77	4.58	6.95	
% FBD	86.03	86.64	92.37	
Poorest/Richest inequalities				
aOR (95% CI) for C-section^a^	0.91 (0.13; 6.27)	13.86 (3.36; 7.30)***	2.45 (1.04; 5.78)*	0.743
aOR (95% CI) for FBD^b^	5.54 (3.32; 9.26)***	2.21 (1.17; 4.16)*	8.58 (5.42; 13.56)***	0.449

*aOR* adjusted odds ratio *C-section* caesarean section, and *FBD* facility based delivery

Period 1 = February 1996 to May 2001; Period 2 = June 2001 to December 2004; and Period 3 = January 2005 to January 2013

*p ≤ 0.05; **p ≤ 0.01; ***p ≤ 0.001; *m marginal level of significance (0.05 < m < 0.10)

^a^The aORs for C-section access were estimated with the use of the multivariate logistic regression models (adjusted based on age, education, wealth quintile of household, zone of residence, parity, and multiple pregnancy, as well as sampling weight, clustering and strata)

^b^The aORs for FBD access were estimated with the use of the multivariate logistic regression models (adjusted based on age, education, wealth quintile of household, zone of residence, parity, and multiple pregnancy, as well as sampling weight, clustering and strata)

^c^*p*-values of the interaction between categories (urban vs rural or educated vs non-educated) and period (period 1 vs period 3) were estimated with the use of the multivariate logistic regression models (adjusted based on age, education, wealth quintile of household, parity, and multiple pregnancy, as well as sampling weight, clustering and strata)

The risk of C-section delivery was higher among educated women than among non-educated women (aOR > 1) irrespective of the period, but these inequalities decreased after implementation of the policy (*p*-value = 0.043). Inequalities linked to maternal education were also significant for FBD, but no significant change was found between periods 1 and 3 (*p*-value = 0.702, Table 2).

We observe inequalities in favour of the richest compared to the poorest in access to C-sections from period 2 and in access to FBD for all periods. No significant change was found between periods 1 and 3. Finally, confirmation of these results is shown in Fig. 3. In Benin, the richer women were, the better access they had to C-sections and FBDs, irrespective of the period.

Although no significant change in wealth-related inequalities related to C-section access was found between periods, we observed a significant decrease in wealth-related inequalities in access to FBD between periods 1 and 2 (*p*-value of the Z-test = 0.000).

For all the regressions performed in Tables 2 and 3, the F-adjusted mean residual goodness-of-fit test was applied, and the results suggested no evidence of lack of fit. No collinearity between the variables was detected.

## Discussion

In both countries, we found no significant change in socioeconomic inequalities in access to C-section and FBD after the introduction of the free C-section policy, except for a reduction in education-related inequalities in access to C-section in Mali.

Otherwise, all significant changes that we observed began prior to the introduction of the policy.

Our results challenge ideas about free healthcare policies for all, which mainly benefit the most favoured social groups, such as the richest people or those who live in urban areas [36]. This study provides evidence that a user fee exemption policy does not necessary lead to an increase in existing inequalities and can benefit the least advantaged population categories. Similar results have already been shown not only in Africa but also in Asia and Latin America [7, 37]. However, even though a free C-section policy does not increase inequalities, these inequalities are still present, and increased health care access is insufficient for the poorest, non-educated and rural women. Some prior studies have already reached the same conclusions [24, 38, 39]. Similar to other reports [40–42], we recommend switching to mechanisms that combine both a universal approach (health care for all) and targeted action for vulnerable populations to address this issue. The goal is to ensure equal health care access across individuals.

Our results on wealth-related inequalities are consistent with the results found by McKinnon et al. [22] concerning user fee removal policies focused on pregnant women (not specifically focused on C-sections) in other sub-Saharan African countries. They did not find robust evidence that this reform was associated with a reduction in wealth-related inequality in access to FBD. We found no difference in the evolution of wealth-related inequalities in access to C-section after implementation of the policy. The free policy benefitted the richest and poorest women in the same way. This conclusion is very similar to findings from another study, which concluded that the user fee policy in Burkina Faso benefited all categories of women, including the poorest women [43].

We found that education-related inequalities related to maternal education in access to C-section decreased in Mali after implementation of the free policy. Education allows women to evaluate whether they require treatment [44]. Prior to implementation of the free policy (when C-sections were more expensive), we hypothesize that educated women tended to pay for this intervention because they were aware of its benefits for themselves and their newborn infant when faced with obstetric complications. In contrast, non-educated women (often the poorest) could not afford to pay for an expensive intervention, regardless of its necessity. After implementation of the reform, even if non-educated women were not sensitive to the role of C-sections, they were more likely to follow their doctor’s decision when the fear of having a financial burden disappeared.

Furthermore, user fee exemptions can contribute to improving the decision-making power of women in health matters [45]. This policy might thus encourage pregnant women to request a C-section.

In Benin, unlike in Mali, the results revealed no significant differences in access to C-sections between urban and rural women or between educated and non-educated women, before or after the implementation of the policy. Benin and its population of 11 million is approximately 10 times smaller in terms of surface area than Mali (population 18 million) (Word Bank data). This difference implies that women have a better access to health care during pregnancy, even if they live in rural areas, as the distance to a health centre is less important.

Furthermore, according to the DHS 2011–12 in Benin, women who gave birth within the last 5 years prior to the interview were more urban (40% vs 19% in Mali) and more educated (28% received a primary education or more vs 16% in Mali) [46, 47]. The only related inequality was that the richest women had greater access to C-sections than the poorest women. Policies must therefore focus on measures that truly eliminate this inequality.

We did not find any studies that confirmed our findings on urban-rural access to C-sections in Africa. This result is an original finding showing that a user fee exemption at the point of service is not enough to reduce urban-rural disparities in access to C-section, even if rural women are often the poorest. Among the approximately 32,469 women living in rural areas who were included in our analysis, 27% belonged to the poorest quintile of wealth, while 49% of urban women were in the richest quintile.

Urban-rural inequalities in Mali decreased between period 1 (the end of the 1990s) and period 2 (the early 2000s). The Reference Evacuation System (RES) launched in 2002 in Mali can explain this decrease. The RES relies on improvements in communication, transportation, community cost-sharing, training and equipment in referral hospitals. A study showed that the RES had a positive impact on C-section rates among rural populations [48].

While the free C-section policy in Mali helped reduce education-related inequalities, some measures taken before the exemption had an effect on the inequalities between urban and rural areas. Mali still needs to focus more on the poorest women to achieve wealth-related equity.

This study has limitations. First, we used DHS data to investigate access to C-sections and FBDs. The reliability of this kind of survey can be questioned as every variable is self-reported and thus potentially subject to possible misclassification and recall bias. Moreover, this kind of survey collects information only on live births, leading to the omission of all stillbirths in these analyses. Stillbirth rates are high in western African countries [49], and there is evidence that C-section rates and intrapartum stillbirth rates are correlated [50]. However, we could not study these cases with our data. Second, this study did not consider the level of implementation of the free C-section policy in both countries. Previous studies showed that the cost of C-sections was still high in many hospitals in Benin after the policy was introduced [15] and that 91% of women still paid for their C-sections in a rural area of Mali during the period 2008–2011 [51]. The impact of this policy on socioeconomic inequalities might have been more important than the observed impact if C-sections were truly free for every woman. Third, DHSs provide information only on whether a child was born by C-section, but we could not verify whether the procedure was required. A recent study showed that C-section rates in Mali and Benin are very high for low-risk women and for women with a previous C-section [52]. These findings suggest that some of these C-sections would not be medically justified. The World Health Organization (WHO) stated in 2015 that “C-sections should be undertaken when medically necessary” [53], and there is no evidence showing a benefit of C-sections for women or infants who do not require this procedure. Fourth, we have only 3 years of data in Benin after the implementation of the policy. This limitation might partly explain why we found no significant evolution of inequalities in Benin after introduction of the policy. The use of the next DHS is necessary to study the sustainability of these impacts.

## Conclusion

This study provides evidence that urban/rural and socioeconomic inequalities in C-section access did not change substantially in Benin or Mali after the countries implemented free C-section policies.

To achieve universal health coverage (UHC) and allow every woman to have access to C-sections without suffering from financial issues, the removal of financial barriers such as user fees must be a priority.

However, to improve equity in access to C-sections, user fee exemptions should not be enacted alone, and a voluntary governmental policy must be established to target vulnerable women without leaving anyone behind.

## Abbreviations

aOR: Adjusted odds ratio
BI: Bamako Initiative
CC: Concentration curve
C-sections: Caesarean sections
DHS: Demographic and Health Survey
FBD: Facility based delivery
RES: Reference Evacuation System
UHC: Universal health coverage
WHO: World Health Organization
